# Chromoblastomycosis Presenting as a Solitary Lesion in a Non-endemic Region

**DOI:** 10.7759/cureus.49791

**Published:** 2023-12-01

**Authors:** Yelena Dokic, Gordana Verstovsek, Theodore Rosen

**Affiliations:** 1 Dermatology, Baylor College of Medicine, Houston, USA; 2 Pathology and Immunology, Michael E. DeBakey Veterans Affairs Medical Center, Baylor College of Medicine, Houston, USA; 3 Dermatology, Michael E. DeBakey Veterans Affairs Medical Center, Baylor College of Medicine, Houston, USA

**Keywords:** copper penny spores, medlar bodies, cutaneous fungal infections, deep fungal infections, dermatology, infectious disease, itraconazole, dematiaceous fungus, chromoblastomycosis

## Abstract

Chromoblastomycosis is a neglected tropical disease typically found in endemic tropical and subtropical regions. Herein, we discuss a rare case of a 55-year-old man in Texas who presented with an exophytic papule on the forearm, diagnosed to have chromoblastomycosis by shave biopsy and subsequent histopathological analysis. Treatment options for chromoblastomycosis include long-term oral antifungal therapy with itraconazole, physical modalities such as heat therapy in conjunction with oral antifungals, and surgical interventions such as cryosurgery or surgical excision.

## Introduction

Chromoblastomycosis is a neglected tropical disease typically found in endemic tropical and subtropical regions such as rural Latin America, the Caribbean, Africa, and Asia [1,2]. It is typically caused by transcutaneous inoculation of the fungus after handling an environmental source, such as soil, tree branches, plant debris, and wood. It is rare for chromoblastomycosis to present in the United States, especially as a solitary papule, but such is the case for our patient. Herein, we present an unusual case of chromoblastomycosis in a 55-year-old man presenting as a single lesion on a patient’s forearm in Texas, a non-endemic region.

## Case presentation

**Figure 1:**
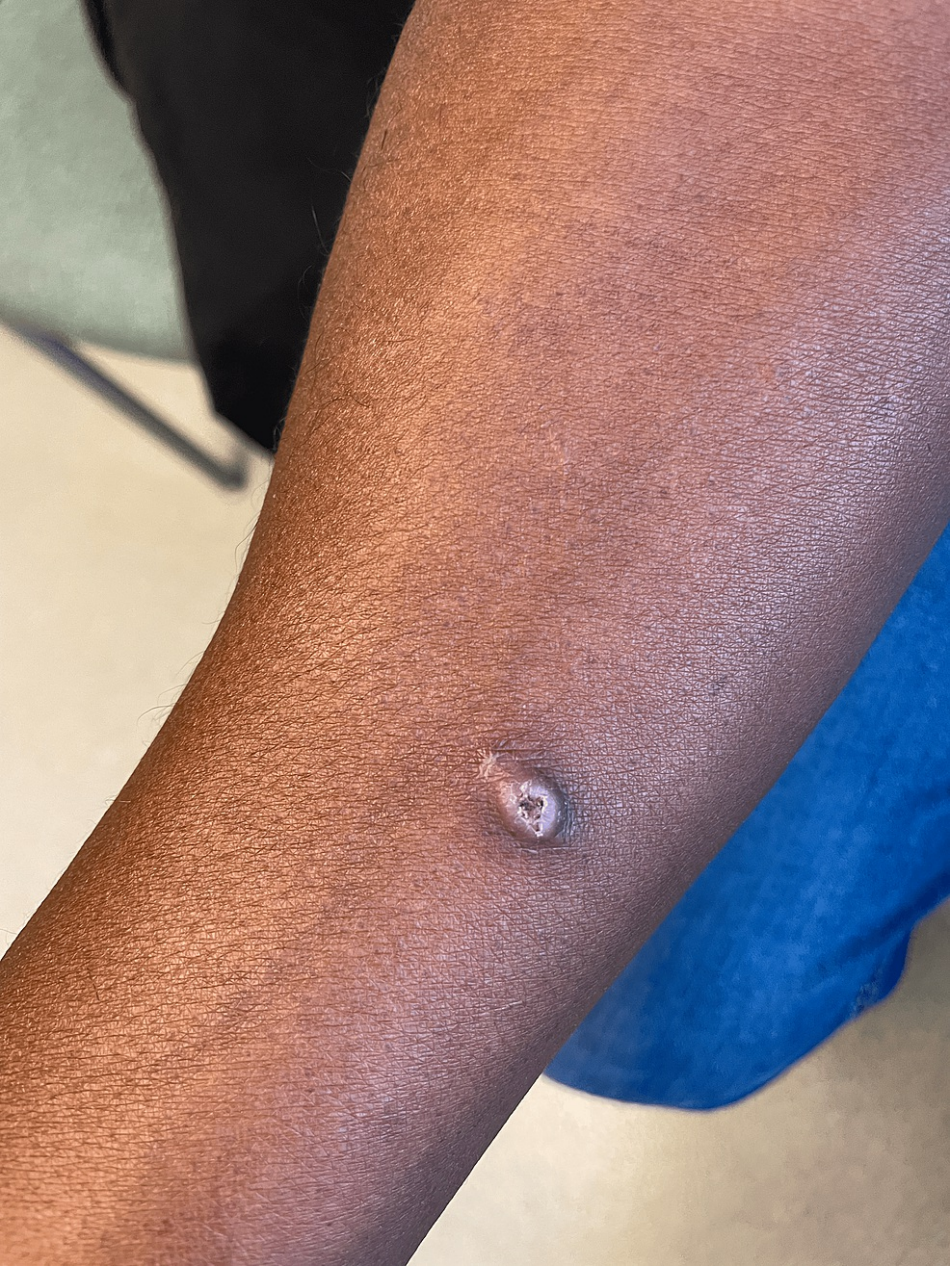
Exophytic papule on the right forearm

A 55-year-old African-American man with well-controlled HIV (most recent CD4 count was 740 cells/µL and most recent viral load was <20 copies/mL detected) and Parkinson's disease presented with a 0.6 cm x 0.8 cm exophytic papule on the forearm with a rolled border and central yellow and red crusting (Figure 1).

The lesion had been present for about one month prior to presentation and was slowly growing. The patient denied any insect bites or previous skin cancers. He is a police officer by occupation and denies traveling outside of Texas. The only traveling he had done was to East Texas two months before the lesion presented on his arm. He stated that he was clearing tree branches and bushes at a house in East Texas just prior to the lesion appearing on his forearm and that he might have suffered penetrating trauma from working in the foliage. The rest of the physical exam was unremarkable.

**Figure 2:**
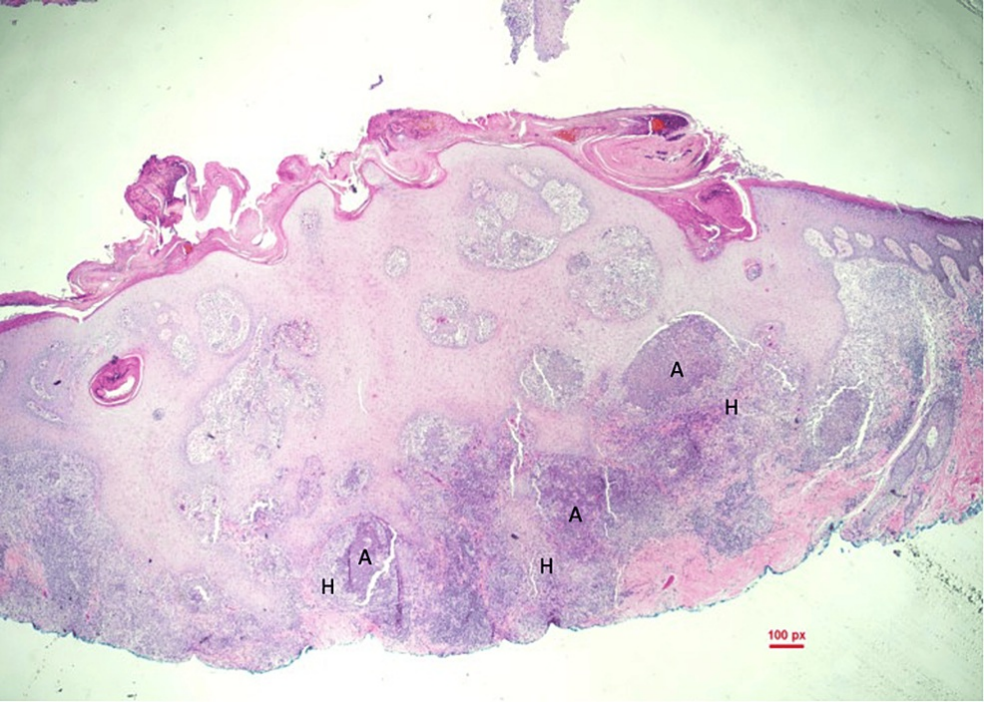
Numerous dermal neutrophilic microabscesses (A) surrounded by epithelioid histiocytes (H) (20x)

**Figure 3:**
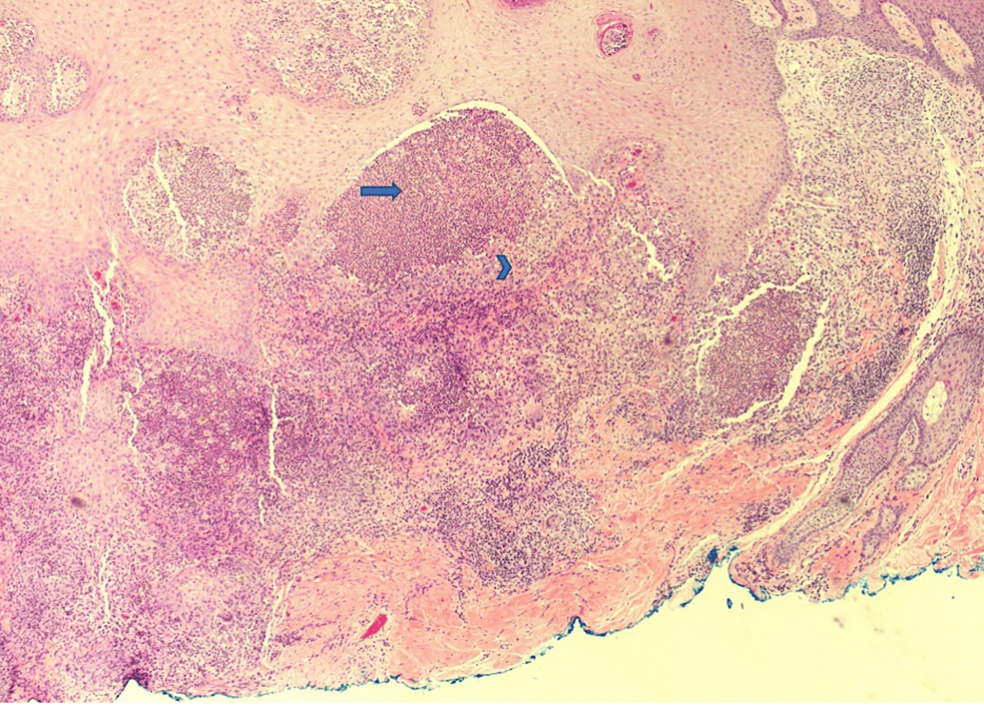
Darker staining microabscesses (arrow) with adjacent paler granuloma (arrowhead) (40x).

**Figure 4:**
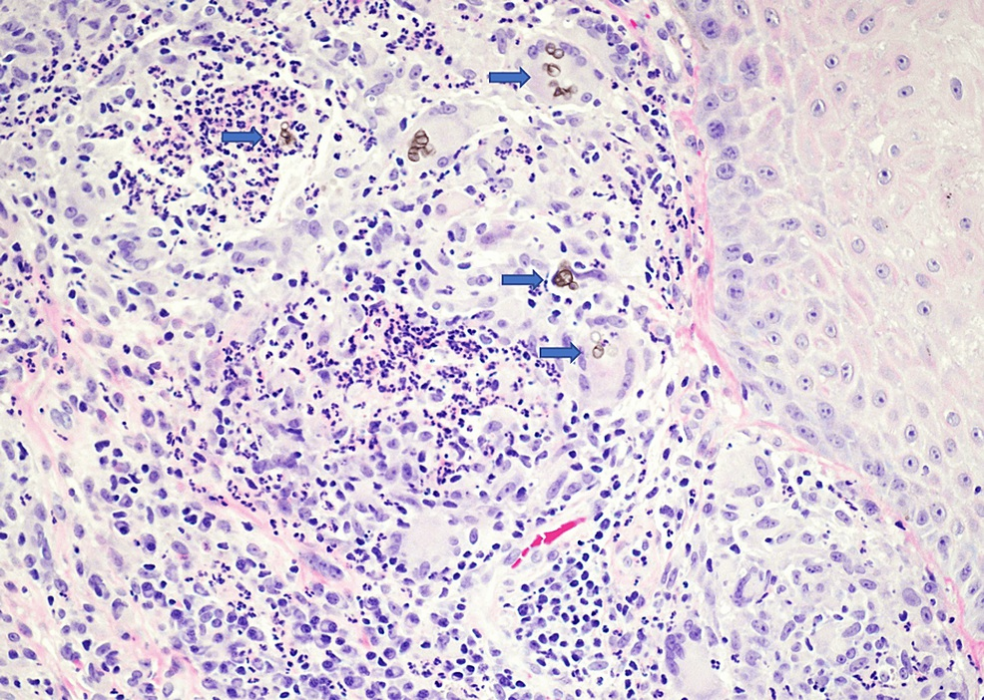
Within the inflammation, Medlar bodies (arrows) are present within giant cells and surrounded by neutrophils (200x)

**Figure 5:**
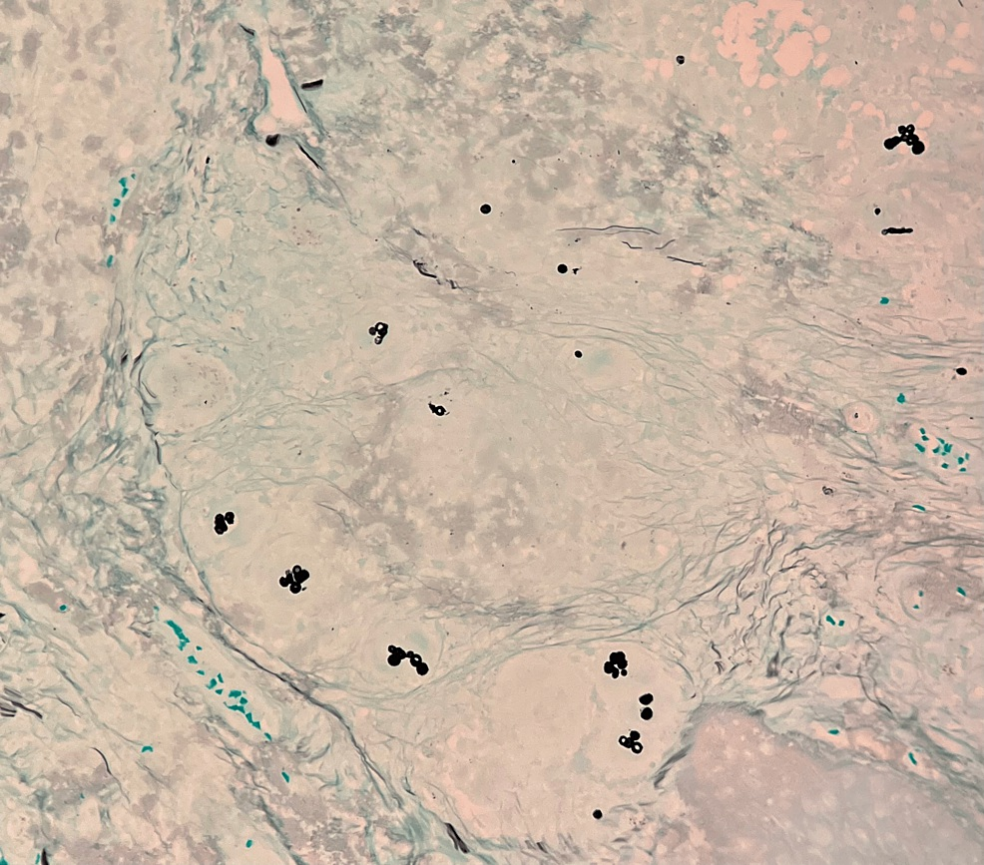
GMS stain highlighting the fungal organisms in black (200x)

The differential diagnosis for the lesion included basal cell carcinoma, squamous cell carcinoma, foreign body granuloma, an infectious process, and other neoplastic or inflammatory processes. A shave biopsy of the papule on the right forearm was conducted, and histopathology revealed pyogranulomatous inflammation associated with pigmented fungal organisms (Figures 2, 3). Within the inflammation, there were numerous brown-pigmented round yeast structures, some showing broad-based budding. The brown structures, also known as Medlar bodies, are often likened to resembling copper pennies (Figure 4). A fungal Grocott-Gömöri's methenamine silver stain was conducted, which further highlighted the fungal organisms (Figure 5). The findings were deemed to be consistent with the presence of dematiaceous fungus, specifically chromoblastomycosis.

The patient was notified regarding the biopsy results and the likely association with his previous yard work. Although the entire lesion was ostensibly removed in the biopsy, he was prescribed itraconazole 200 mg daily for two months as a prophylactic measure. It was also recommended that he start applying a heating pad to the biopsy site three times daily for 10-15 minutes for two months, while on the itraconazole.

## Discussion

Chromoblastomycosis is a granulomatous fungal infection of the skin and subcutaneous tissue that is caused by dematiaceous fungi, primarily of the genera Fonsecaea, Phialophora, and Cladophialophora [1,2]. Endemic to tropical and subtropical regions, it is rarely found in the United States.

The exact pathogenesis of chromoblastomycosis can be via direct percutaneous inoculation, inhalation, and hematogenous dissemination. The fungus is found in soil, plants, and decaying wood. If organic material that is contaminated by the fungal organism is implicated in a traumatic injury, then percutaneous implantation of the fungus may occur [3].

Once inoculated into the skin, chromoblastomycosis is a locally progressive disease and can continue to grow over many years. Chromoblastomycosis can have a wide variety of morphologies [4]. It classically presents on the lower extremities of middle-aged men working in rural areas of tropical climates as verrucous reddish-purple papules. The lesions can be painless or mildly pruritic. Weeks to months later, similar lesions tend to appear peripherally, with the lesions growing centrifugally. Late lesions can appear as annular with central scarring. If the lesions are left to continue growing, entire limbs can become involved, covered with large, vegetative, verrucous plaques. Although the disease tends to primarily grow locally, satellite lesions can occur rarely, which are thought to spread via lymphatics.

The diagnosis of chromoblastomycosis is confirmed by histopathological examination of skin biopsy specimens, which typically reveal muriform fungal cells (Medlar bodies) within the granulomatous infiltrate. Fungal culture may also be helpful in identifying the causative fungal species.

Therapeutic options for chromoblastomycosis center around oral antifungal agents, such as itraconazole, terbinafine, or posaconazole. Long-term itraconazole treatment (200-400 mg daily) is the first-line therapy, with a duration ranging from several months to even years, depending on the clinical severity [5]. Terbinafine (250-500 mg daily) serves as a second-line treatment. Posaconazole (400 mg twice a day) may be considered for refractory cases, but its high cost limits its widespread use. Although fluconazole has one of the highest in vitro minimum fungicidal concentrations for the causative species of chromoblastomycosis, it may be used at 300 mg weekly or 150 mg daily. Alternating weeks between daily itraconazole and daily terbinafine therapy (itraconazole 200-400 mg/day and terbinafine 500-750 mg/day) is also an option [5].

Heat therapy (local surface temperature of 46°C) is often used in conjunction with oral antifungals, as it has proven effective in causing involution of the fungal lesions [6].

Cryosurgery, involving the application of liquid nitrogen to freeze and destroy the affected tissue, can be used in combination with oral antifungals as a possible treatment for chromoblastomycosis [7]. This option is more effective for smaller lesions, fewer lesions, and lesions that are not in anatomically sensitive areas. It should be avoided in areas with skin folds to prevent secondary fibrosis and scarring [7]. Counseling skin of color patients regarding the possible side effects of hypopigmentation following cryotherapy is also important.

Surgical excision with appropriate margins is recommended for small, early lesions to facilitate complete removal of the fungal elements and prevent further spread. Curettage with electrodessication is generally not advised, as it may promote the lymphatic spread of the infection [8]. Other potential destructive methods include photodynamic therapy (PDT), CO_2_ laser photocoagulation, and immunoadjuvants such as topical imiquimod [5].

Despite the challenges in treating chromoblastomycosis, early diagnosis, and appropriate management can lead to significant improvement and even complete resolution of the disease.

## Conclusions

Our patient was a 55-year-old man in Texas who presented with an exophytic papule on the forearm and was diagnosed with biopsy-proven chromoblastomycosis. Several treatment modalities exist, and our patient is currently receiving itraconazole treatment with heating pads. Although rare, chromoblastomycosis can present as a solitary papule in the United States, a non-endemic region.
